# LncRNAs as novel players in hepatocellular carcinoma recurrence

**DOI:** 10.18632/oncotarget.26202

**Published:** 2018-10-12

**Authors:** Laura Gramantieri, Michele Baglioni, Francesca Fornari, Maria Antonella Laginestra, Manuela Ferracin, Valentina Indio, Matteo Ravaioli, Matteo Cescon, Vanessa De Pace, Simona Leoni, Camelia Alexandra Coadă, Massimo Negrini, Luigi Bolondi, Catia Giovannini

**Affiliations:** ^1^ Center for Applied Biomedical Research, St. Orsola-Malpighi University Hospital, Bologna, Italy; ^2^ Department of Medical and Surgical Sciences, DIMEC, University of Bologna, Bologna, Italy; ^3^ Department of Experimental, Diagnostic and Specialty Medicine, University of Bologna, Bologna, Italy; ^4^ “Giorgio Prodi” Cancer Research Center, University of Bologna, Bologna, Italy; ^5^ General Surgery and Transplant Unit, Sant’Orsola-Malpighi University Hospital, Bologna, Italy; ^6^ Department of Morphology, Surgery and Experimental Medicine, University of Ferrara, Ferrara, Italy

**Keywords:** hepatocellular carcinoma, LUCAT1, CASC9, biomarkers

## Abstract

Long non-coding RNAs (lncRNAs) are ncRNAs more than 200 nucleotides long that participate to a wide range of biological functions. However, their role in cancer is poorly known. By using an NGS-based approach we analyzed the intragenic and poliA-lncRNAs in hepatocellular carcinoma (HCC) and we assayed the relationships between their deregulated expression and clinical-pathological characteristics. The expression profile of lncRNAs was studied in a discovery series of 28 HCC and matched cirrhosis and was validated in an independent cohort of 32 HCC patients both in tissue and serum. The correlation between lncRNA expression and clinical-pathological variables, EMT markers and putative sponged microRNAs level were investigated. Functional experiments were performed in HCC-derived cell lines to clarify the role of selected lncRNAs in HCC. A panel of deregulated lncRNAs differentiated HCC from cirrhotic tissue. CASC9 and LUCAT1 were up-regulated in a subset of HCC-derived cell lines and in half of HCCs which displayed a lower recurrence after surgery. LUCAT1 and CASC9 silencing increased cell motility and invasion capability in HCC cells and influenced the EMT phenotype. LUCAT1 was demonstrated to directly sponge the onco-miR-181d-5p. Both LUCAT1 and CASC9 were secreted in exosomes, and higher circulating CASC9 levels were associated with tumor size and HCC recurrence after surgery, suggesting its potential usage as putative non-invasive prognostic biomarker of recurrence.

## INTRODUCTION

The non-protein coding portion of the transcriptome has recently gained a prominent relevance due to the increasing evidence of its role in the development and progression of a wide range of diseases. Non-coding RNAs (ncRNAs) comprise several classes of RNAs including small ncRNAs (microRNAs, PIWI-interacting RNAs, small nucleolar RNAs) and the heterogeneous group of long non-coding RNAs (lncRNAs) classified as ncRNAs longer than 200 nucleotides. While microRNAs deregulation is now considered a hallmark of human cancers, including hepatocellular carcinoma (HCC) [1], little is known about the role of lncRNAs in tumorigenesis. Physiologically, lncRNAs participate to gene expression modulation at multiple levels, from transcription and translation regulation to control of mRNA stability and protein degradation [2]. Recent reports outline the role of specific lncRNAs in liver carcinogenesis by regulating crucial molecular pathways such as the Wnt-Beta Catenin and the STAT3 ones and, as such, they were proposed as possible novel biomarkers and therapeutic targets [3, 4]. Among the most studied lncRNAs in HCC, HOTAIR (Hox transcript antisense intergenic RNA) was shown to have oncogenic functions [5] and its overexpression was associated with higher HCC recurrence after surgery and higher frequency of lymph node metastasis. HULC, DILC, PVT1 and MALAT1 were shown to participate to hepatocarcinogenesis and were suggested as putative biomarkers [6–9]. Klingenberg et al recently revised the main findings accumulated so far on lncRNAs deregulated expression in liver cancer development and progression, their relevance in the modulation of HCC-related molecular pathways as well as their possible roles as biomarkers [10]. Due to their secretion into body fluids, specific lncRNAs were proposed also as non-invasive biomarkers in patients with HCC [11]. Here, we focused our attention on intragenic and polyA lncRNAs emerged from a RNA sequencing study conducted on matched HCC and liver cirrhosis (LC). Data obtained from a discovery setting were validated in an independent series of patients. The association between aberrant expression of a panel of lncRNAs and HCC recurrence was identified and mechanistic insights were produced in HCC-derived cell lines supporting their role in cell motility, invasion capability and miRNA sponge function. To explore their role as putative non invasive biomarkers, CASC9 overexpression was confirmed in serum of HCC patients. High CASC9 serum levels at the time of surgery were associated with the subsequent development of HCC recurrence, envisaging a putative role as non-invasive prognostic biomarkers.

## RESULTS

### LncRNA profiling

**Figure 1:**
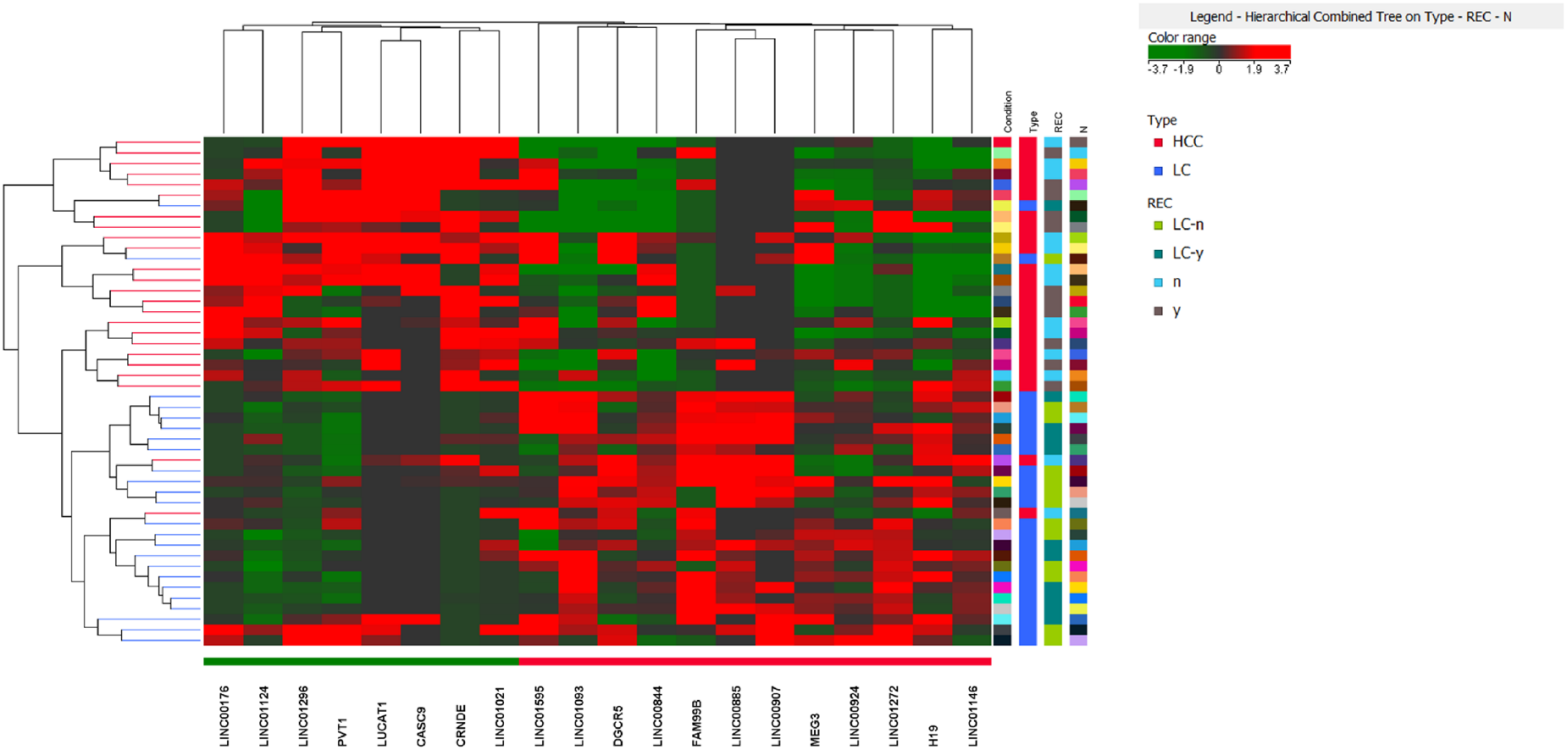
Heat map of long non-coding RNA differentiating HCC from LC adopting a FC>2 (adjusted p<0. 05)

**Figure 2:**
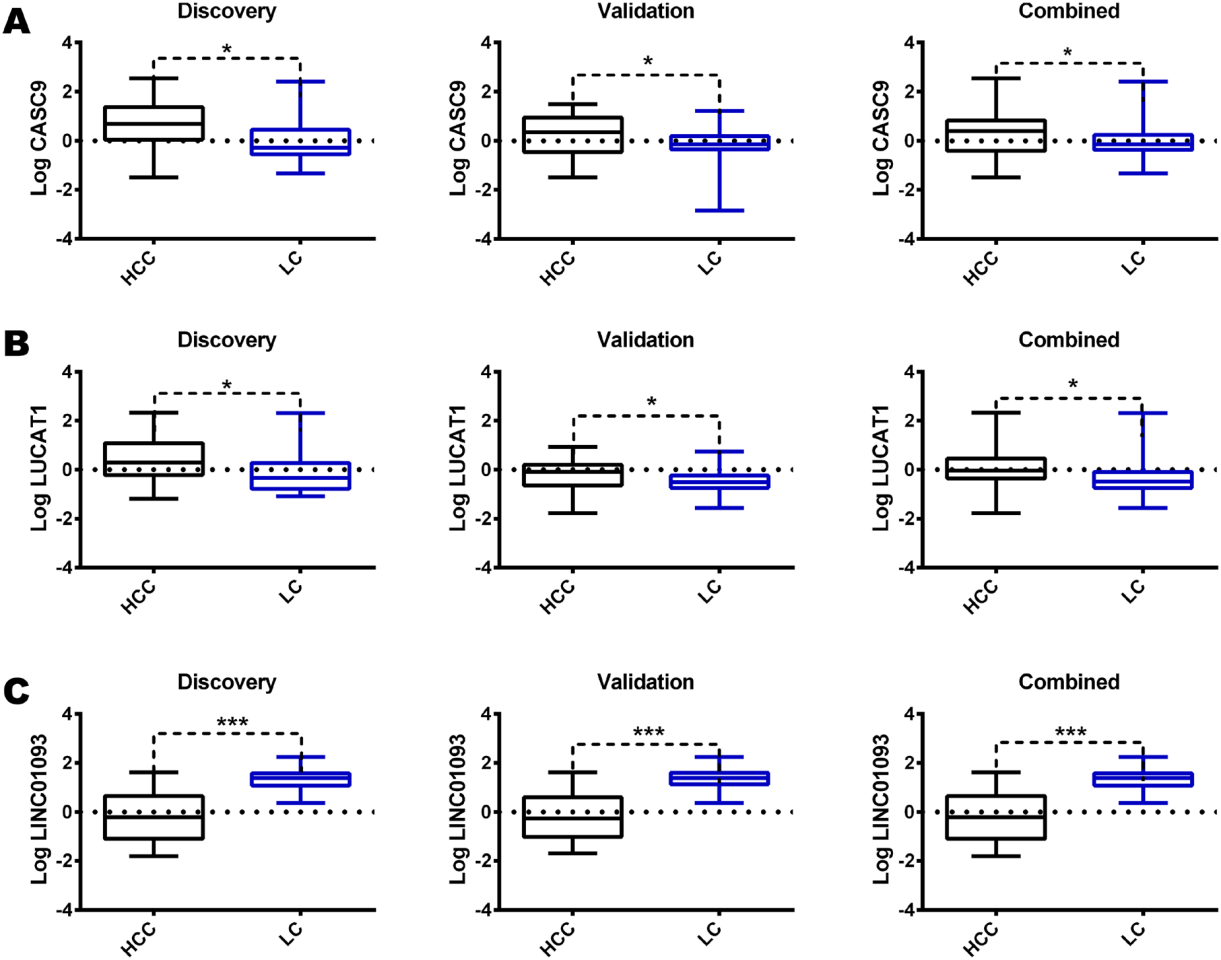
Validation of RNAseq results Box plot graphic representation of CASC9 **(A)**, LUCAT1 **(B)** and LINC01093 **(C)** expression in matched LC and HCC tissue in the discovery, validation, and whole cohorts. lncRNAs were tested by Real-Time RT-PCR. Logarithmic transformation was applied to Real-Time RT-PCR data. Difference in expression levels were confirmed as statistically significant (^*^P<0.05; ^***^P<0.001) by two tailed student's t test.

**Figure 3:**
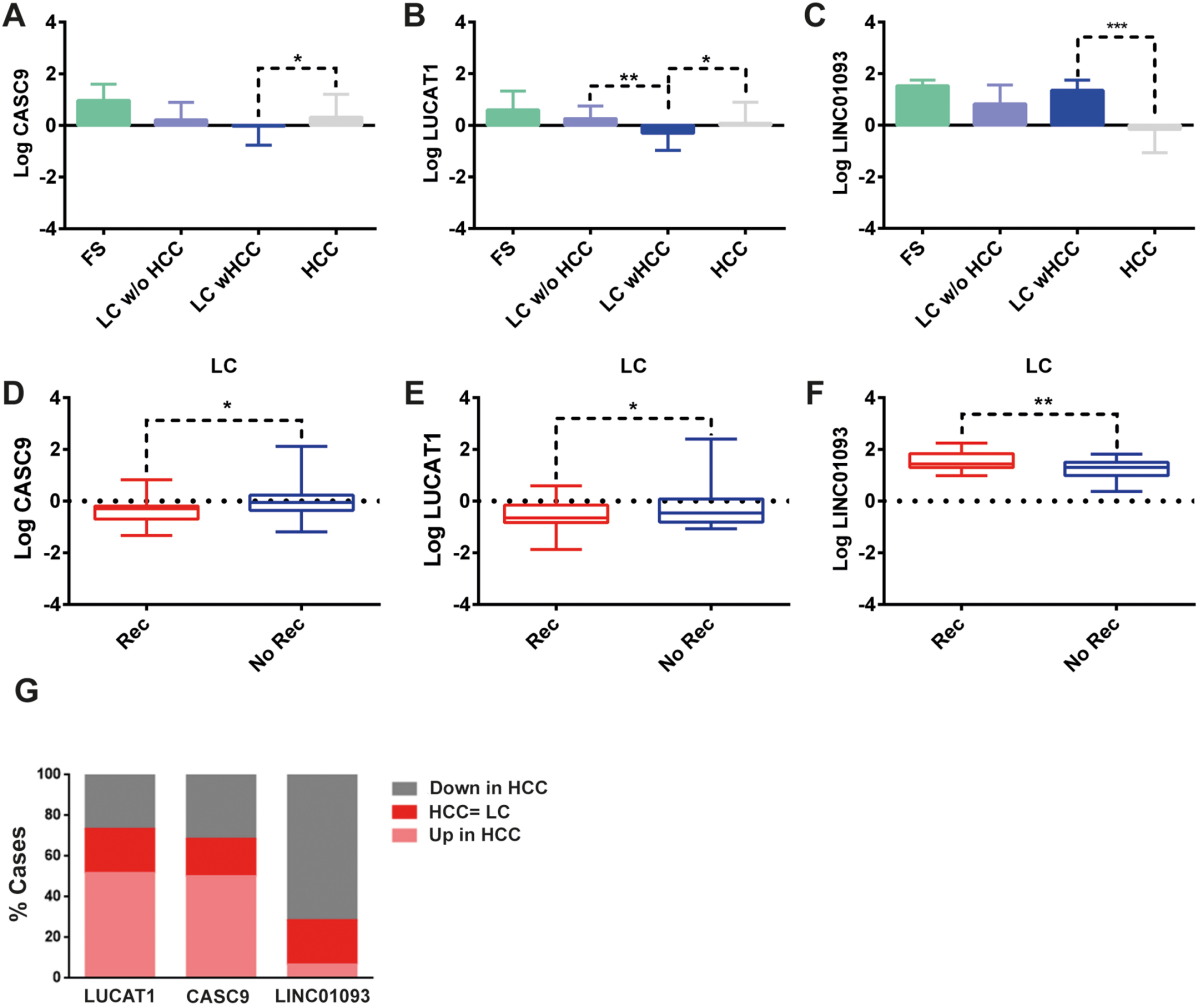
Role of lncRNAs in the field effect Box plot graphic representation of CASC9 **(A)**, LUCAT1 **(B)** and LINC01093 **(C)** expression in healthy liver tissue (FS), LC undergoing orthotopic liver transplantation (OLT) without HCC (LC w/o HCC), LC tissue in patients with concomitant HCC (LC wHCC) and HCC tissues in the whole cohort of 60 patients. LncRNAs expression was also analyzed in LC tissues surrounding HCC **(D-F)** comparing cases with poor (<2 years, N.=34 patients) vs. good (>2 years, N.=26 patients) recurrence-free survival. Logarithmic transformation was applied to Real-Time RT-PCR data. GAPDH was used as housekeeping gene. Difference in expression levels were confirmed as statistically significant for LUCAT1 when comparing liver cirrhosis with vs without HCC. In addition, cirrhotic tissues from patients with and without HCC recurrence two years after surgery showed different expression levels of all three lncRNAs. **(G)** HCC distribution (60 cases) according to up-regulation, down-regulation or similar expression of LNCRNAs in HCC tissues when compared with surrounding liver. ^*^P<0.05; ^**^P<0.01; ^***^P<0.001 (by two tailed student's t test).

We performed a whole transcriptome analysis, including intragenic and polyA lncRNAs, in a discovery cohort of 28 matched HCC and LC by using a RNA-sequencing approach. We detected the expression of 14294 genes, including 136 non-protein coding transcripts. We focused our attention on the lncRNAs whose expression profiles differentiated HCC from LC (Figure 1). We identified a panel of 18 lncRNAs that significantly differentiate HCC from cirrhosis with 9 up-regulated and 9 down-regulated lncRNAs in HCC tissues. Among the deregulated lncRNAs, LUCAT1 and CASC9 were validated by Real-Time RT-PCR in the same discovery cohort and then in an independent cohort of 32 clinically annotated patients surgically treated for HCC (validation set) due to their relevant increase in a subset of HCCs. NGS data were confirmed in both the discovery and validation cohorts (Figure 2). Among the 60 HCC cases, CASC9 was up-regulated in 30 cases (with a down-regulation in 19 cases and absence of variation in 11), LUCAT1 was up-regulated in 31 (with a down-regulation in 16 cases and absence of variation in 13). Conversely, LINC01093 was strongly down-regulated in 71.6% of HCCs. An impressive correlation between LUCAT1 and CASC9 expression was found both in HCC (Pearson's correlation 0.985; p<0.0001) and cirrhotic tissue (Pearson's correlation, 0.959; p<0.0001). To further evaluate the modulation of CASC9, LUCAT1 and LINC01093 expression across the progression of chronic liver disease towards HCC, the expression levels of these lncRNAs were also tested in healthy liver and cirrhosis without or with synchronous HCC, obtained from explanted livers. As shown in Figure 3A-3B, a trend towards a decrease of CASC9 and LUCAT1 was observed, starting from healthy liver, to cirrhosis without HCC, to cirrhosis complicated by HCC with LUCAT1 displaying a relevant down-regulation in cirrhosis complicated by HCC, in line with its possible contribution to hepatocarcinogenesis. The down-regulation of LINC01093 characterizes the majority of HCCs and it appears to provide a promising diagnostic biomarker (Figure 3C). A higher recurrence rate was observed in cases down-regulating LUCAT1 and CASC9 in cirrhotic tissue at the time of surgery (Figure 3D-3E). Unexpectedly, LINC01093 expression was higher in cirrhosis of recurrent patients (Figure 3F). Concerning HCC tissue, the up-regulation of LUCAT1 and CASC9 characterizes 50% of HCCs only. Indeed, 26.7% and 31.7% of cases respectively down-regulate LUCAT1 and CASC9 and another 18-21% of cases do not display any change (Figure 3G). Since LUCAT1 and CASC9 up-regulation segregates in the same subgroup of HCCs, this event might reflect a distinct molecular background driving these changes.

### CASC9 and LUCAT1 up-regulation is associated with lower recurrence in HCC

**Figure 4:**
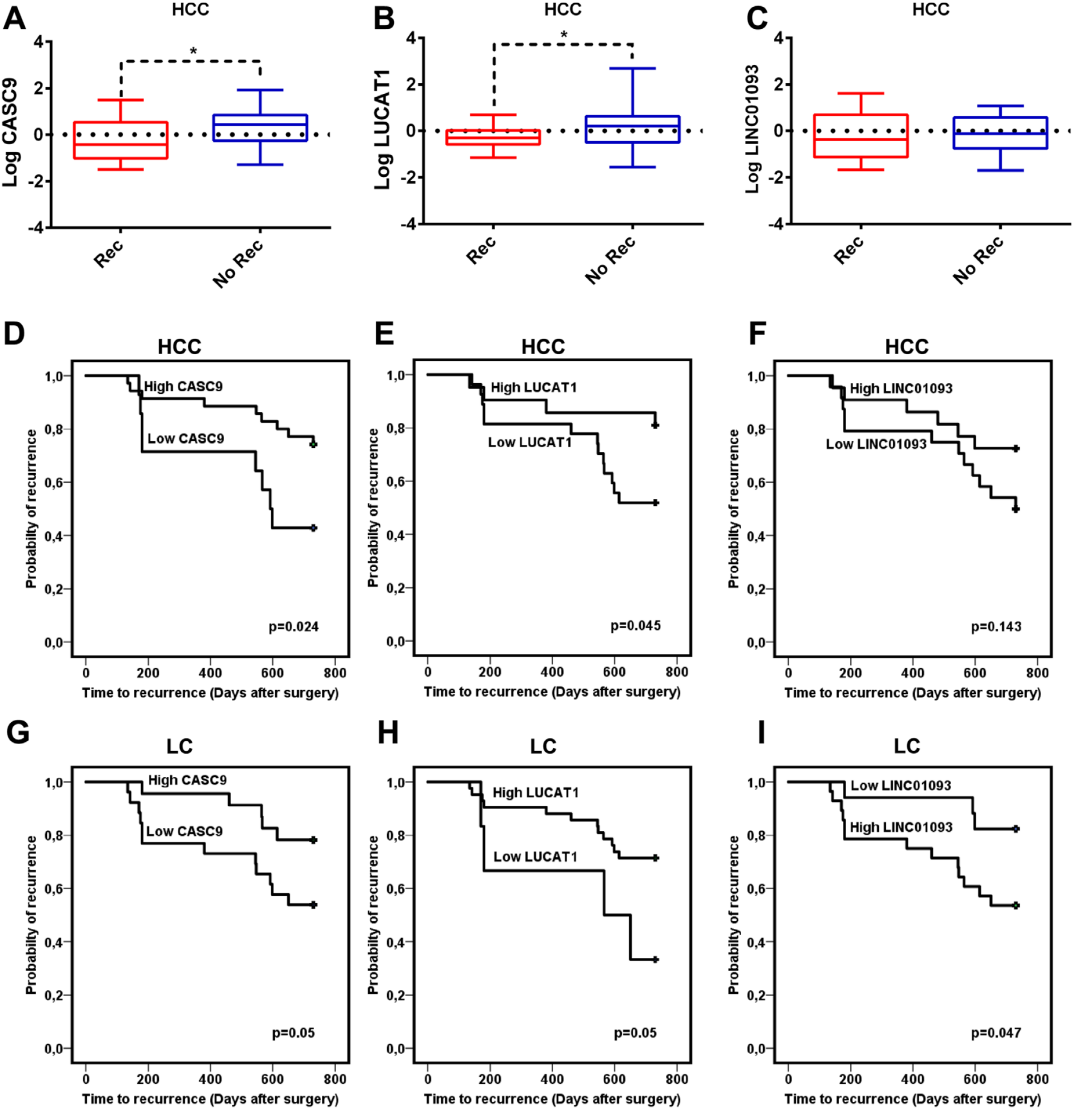
Role of lncRNAs in the recurrence after surgery Box plot graphic representation of CASC9 **(A)**, LUCAT1 **(B)** and LINC0193 **(C)** expression in HCC tissues from patients with a different recurrence rate two years after surgery. Logarithmic transformation was applied to Real-Time RT-PCR data. Difference in expression levels were confirmed as statistically significant for CASC9 (t-test, p=0.02) and LUCAT1 (t-test, P=0.04) while no difference was found for LINC01093. D-I: Kaplan-Meier plot calculated categorizing patients according to lncRNAs deregulated expression in HCC **(D, E, F)** and LC **(G, H, I)** tissues. The up-regulated expression of CASC9 (D) and LUCAT1 (E) in HCC and cirrhotic tissues (G-H) is associated with a longer time to recurrence (TTR) while lower LINC01093 levels in cirrhosis are associated with a longer TTR (I).

To start understanding the roles of deregulated expression of these lncRNAs in HCC and cirrhosis, we evaluated the associations between LUCAT1 and CASC9 expression and clinical-pathological variables. Etiology of the chronic liver disease, histopathological grading, tumor nodularity, size, AFP serum levels and time-to-recurrence (TTR) after surgery were tested against LUCAT1, CASC9 and LINC01093 expression. Higher LUCAT1 and CASC9 expression levels in HCC tissues were associated with a lower recurrence rate in the whole cohort of patients surgically treated for HCC (Figure 4A, 4B) while no difference was observed for LINC01093 (Figure 4C). Kaplan-Meier plot was calculated categorizing patients according to lncRNAs deregulated expression and TTR. The up-regulated expression of LUCAT1 and CASC9 in HCC tissue is associated with a longer TTR after surgery (Figure 4D, 4E), while it was not significant for LINC01093 (Figure 4F). The same findings apply to the deregulated expression of LUCAT1 and CASC9 in the cirrhotic tissues (Figure 4G, 4H) with an association between higher expression levels and a longer TTR. An opposite behavior was observed in the case of LINC01093 (Figure 4I). Multivariate analysis showed AFP (p<0.0001), vascular invasion (p<0.0001) and up-regulation of CASC9 expression in HCC (p<0.0001) as independent predictive factors of recurrence. No association was found between OS and LUCAT1, CASC9 and LINC01093 tissue expression, however this analysis might be not fully reliable due to different therapeutic approaches applied to recurrent disease.

### LUCAT1 and CASC9 contribute to EMT phenotype in HCC

**Figure 5:**
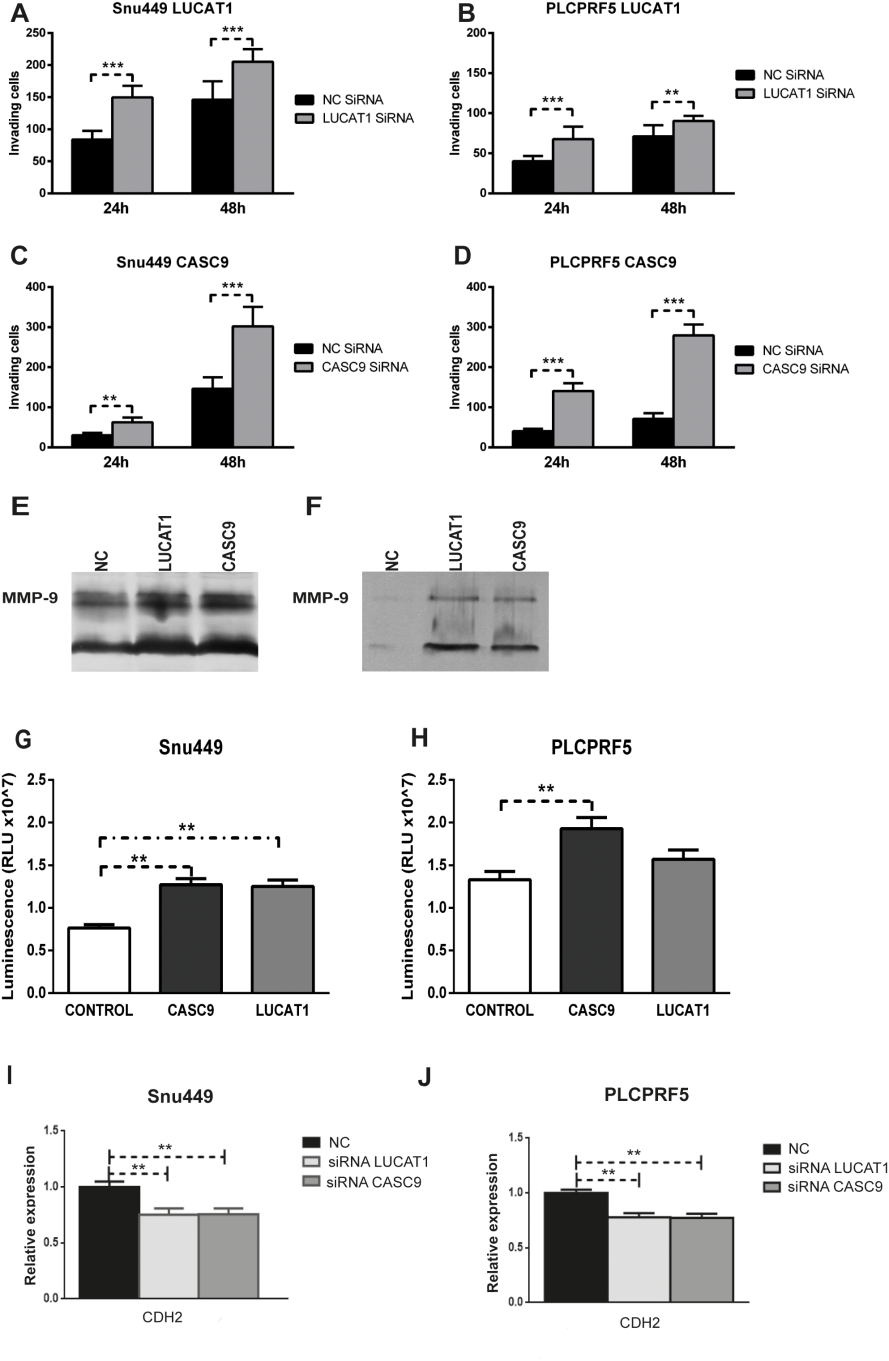
CASC9 and LUCAT1 are involved in cancer cells invasion **(A, B)**:Increased number of invading cells (assayed by Boyden blind-well chambers containing poly-vinyl-pyrrolidone–free polycarbonate filters, coated with Matrigel) at 24 and 48 hours following LUCAT1 inhibition in Snu449 and PLC/PRF5 cells and **(C, D)** following CASC9 inhibition. **(E, F)**: Gelatin zymography assay of metalloproteinase 9 in Snu449 cells (E) and in PLC/PRF5 cells (F) following LUCAT1 and CASC9 inhibition. Lane 1: negative controls; Lane 2: cells treated by LUCAT1 siRNAs; Lane 3: cells treated by CASC9 siRNAs. **(G, H)**: Cell Titer assay in Snu449 and PLC/PRF5 silenced for CASC9 and LUCAT1. Difference in luminescence levels were confirmed as statistically significant (^**^P<0.01) by two tailed Student's t-test. **(I** and **J)**: CDH2 changes following LUCAT1 and CASC9 inhibition in Snu449 and PLC/PRF5 cells.

As shown above, higher LUCAT1 and CASC9 expression in HCC and cirrhotic tissues was associated with a lower recurrence and a longer TTR in patients surgically resected for HCC. We thus assayed *in vitro* the role of LUCAT1 and CASC9 inhibition in cell invasion capability. Five HCC-derived cell lines (HepG2, Huh7, PLC/PRF5, SNU449, Hep3B) were tested for CASC9 and LUCAT1 expression (Supplementary Figure 1A, 1B). PLC/PRF5 and SNU449 cells, reflecting the subgroup of HCCs with both LUCAT1 and CASC9 up-regulated expression, were chosen for *in vitro* assays. These cell lines, and especially SNU449 cells, were also chosen due to their relevant basal invasion capability. SiRNA-mediated partial inhibition of CASC9 and LUCAT1 performed in both cell lines (Supplementary Figure 1C) determined an increased invasion capability, assayed by Boyden blind-well chambers (Figure 5A-5D) which was confirmed by zymography assay of metalloproteinase 9 both in SNU449 cells (Figure 5E) and in PLC/PRF5 cells (Figure 5F). We next ruled out a possible interference on migration assay by a higher proliferation rate following CASC9 and LUCAT1 inhibition. Cell cycle analysis was performed and didn't show any significant change in cell cycle progression after lncRNAs inhibition in both cell lines, suggesting that invasion capability assessment in silenced cells is not invalidated by increased cell proliferation. In addition, CellTiter viability assay resulted significantly higher in silenced SNU449 and PLC/PRF5 cells (Figure 5G, 5H) indicating that, in the absence of cell cycle variations, a higher metabolic activity revealed by increased ATP levels, might be associated with migration activity as previously reported [12, 13]. The association between high LUCAT1 and CASC9 expression and reduced HCC recurrence as well as reduced cell invasion capability, let us to hypothesize a possible association with the EMT phenotype. We thus tested a panel of biomarkers of EMT in HCC cell lines as well as in HCC tissues (60 patients), including CDH1 (E-cadherin), CDH2 (N-Cadherin), CTNNB1 (beta-catenin), TCF7L2 (wnt signaling), VIM (Vimentin), ZEB1, ZEB2, TWIST, SNAIL1 and SNAIL2. We found that both LUCAT1 and CASC9 positively correlated with CDH1 (LUCAT1/CDH1: Pearson's correlation 0.37; p=0.015; CASC9/CDH1: Pearson's correlation 0.46, p=0.001) and a positive correlation was also found between CASC9 and CDH2 (Pearson's correlation 0.44, p=0.003). LINC01093 also positively correlated with CDH1 expression (Pearson's correlation 0.32, p=0.0001). Interestingly, CDH1 and CDH2 up-regulation in HCC tissues was associated with a lower recurrence rate and a longer TTR (Supplementary Figure 2A, 2B) as observed for CASC9 and LUCAT1. In line with these findings, LUCAT1 and CASC9 silencing in HCC cells, was associated with a reduction of CDH2 in both PLC/PRF5 and SNU449 cells (Figure 5I and 5J). Conversely, CDH1 levels could not be reliably assessed due to their constitutive low expression in the tested cell lines. No correlation was found between LUCAT1 and CASC9 expression and VIM, ZEB1, ZEB2, TWIST, SNAIL1 and SNAIL2 expression. Similarly, no correlation was found for TCF7L2, a wnt-related transcription factor predicted to regulate LUCAT1 transcription, as well as for CTNNB1. Accordingly, we did not find any change of beta-catenin expression and localization following LUCAT1 and CASC9 inhibition in PLCPRF5 and SNU449 cells (Supplementary Figure 3).

### LUCAT1 sponges miR-181d-5p

**Figure 6:**
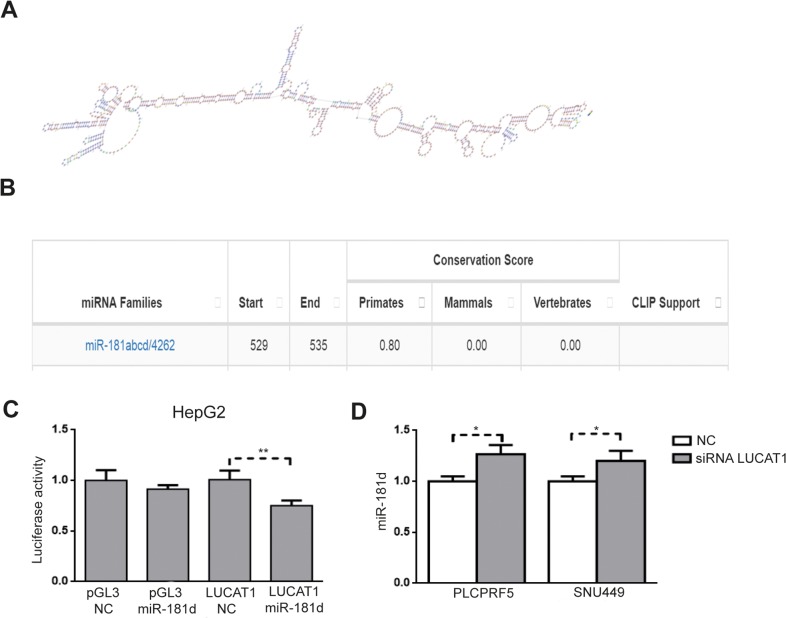
LUCAT1 sponges miR-181d-5p **(A)** LUCAT1 secondary structure (from Annolnc) and **(B)** predicted miR-181 sponging sequence (from Annolnc). **(C)** Luciferase reporter assay in HepG2 cells confirming miR-181d -5p sponging by LUCAT1. **(D)** miR-181d-5p up-regulation following LUCAT1 inhibition in PLCPRF5 and SNU449 cells. ^*^P<0.05; ^**^P<0.01 (by two tailed student's t test).

As a further element shedding light into the molecular mechanisms linking lncRNAs aberrant expression and increased HCC recurrence rate, we tested whether any microRNA with a known role in HCC could be potentially sponged by LUCAT1 and CASC9. MiR-181c-5p and miR-181d-5p resulted putative LUCAT1 targets (Figure 6A and 6B) according to Annolnc (http://annolnc.cbi.pku.edu.cn) a bioinformatics algorithm that predicts the specific binding of microRNAs and lncRNAs on the basis of sequence complementarity [14]. Similarly, miR-145-5p was predicted as a putative target of CASC9. We focused on these microRNAs due to their mechanistic involvement in HCC and to the suggested potential of miR-181 family as biomarkers of an aggressive subgroup of HCCs [15]. MiR-181c/d-5p expression was assessed in primary tumors and a negative correlation was found between LUCAT1 and miR-181d-5p (Pearson's correlation -0.324; p=0.002), while no correlation was found in the case of miR-181c. No correlation was found between CASC9 and miR-145, even though the very low expression levels of miR-145-5p might reduce the performance of this analysis. Due to the negative correlation between LUCAT1 and miR-181d-5p in primary HCCs, we next performed a Luciferase reporter assayin HepG2 cells to verify the direct lncRNA/miRNA interaction. MiR-181d-5p co-transfection determined a 20% decrease of the luciferase activity of pGL3-LUCAT1 vector with respect to negative control co-transfected cells (Student's t-test, p=0.001), demonstrating a sponge effect exerted by LUCAT1 on miR-181d-5p in HCC cells (Figure 6C). Despite statistical significance, the entity of luciferase assay was not so strong as that obtained in miRNAs/mRNAs interactions, likely due to the complex secondary structure of LUCAT1. We next assayed miR-181d-5p changes following LUCAT1 inhibition in PLCPRF5 and SNU449 cells obtaining a slight increase of miR-181d-5p levels (t-test, p<0.05) in both cell lines (Figure 6D). These findings support a sponge function exerted by LUCAT1 on miR-181d-5p, contributing to reduce miR-181d-5p levels in HCCs overexpressing LUCAT1.

### LncRNAs expression in serum and serum exosomes of HCC patients

**Figure 7:**
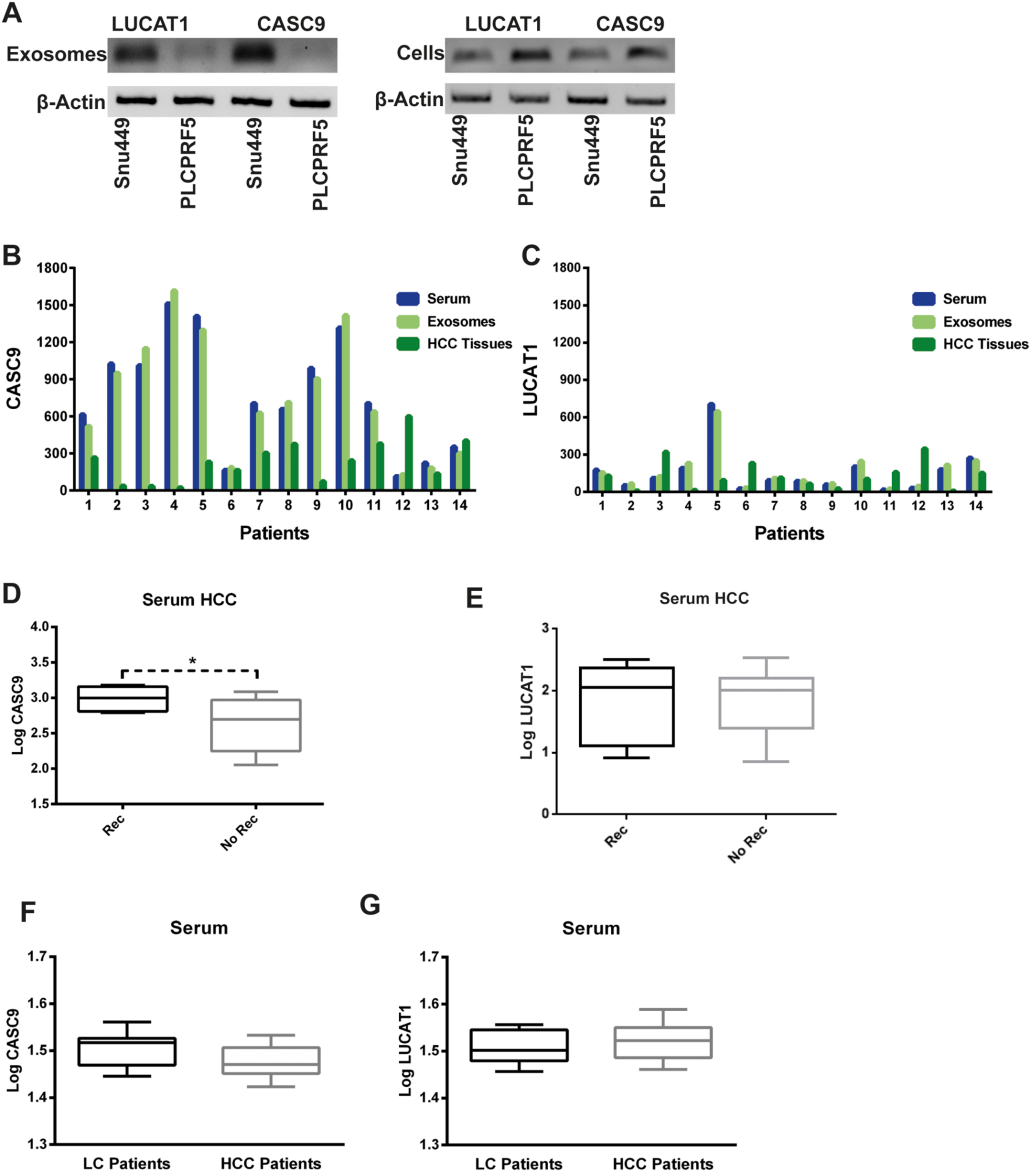
CASC9 and LUCAT1 are detected in serum of patients **(A)**: PCR assay for LUCAT1 (first two lanes) and CASC9 (third and fourth lanes) expression in exosomes (upper panel) and in the intracellular compartment (lower panel). Beta-actin was used as housekeeping for both cells and exosomes. **(B, C)**:CASC9 and LUCAT1 expression in whole serum, serum exosomes and HCC tissue from 14 patients surgically resected for HCC. Beta actin was used as housekeeping for all normalizations and expression data were calculated with 2^-ΔΔCt^ method. **(D, E)**: difference in CASC9 and LUCAT1 serum levels in HCC patients displaying a disease recurrence two years after surgery when compared with patients without HCC recurrence. **(F, G)**: circulating levels of both LUCAT1 and CASC9 in cirrhotic patients without HCC compared with cirrhotic patients with HCC. ^*^P<0.05 (by two tailed student's t test).

Previous evidences outlined the exosome-mediated secretion of lncRNAs and their detection in serum of cancer patients [16], thus we tested HCC derived cell lines for CASC9 and LUCAT1 expression in the intra and extracellular compartment, as well as in the exosomal fraction of cell culture supernatant. SNU449 and PLC/PRF5 were chosen due to their constitutive expression of both LUCAT1 and CASC9 (Supplementary Figure 1). Both CASC9 and LUCAT1 were found in the exosomal fraction of cell culture supernatant, in a relevant amount in the case of SNU449 cells (Figure 7A), suggesting the relevance of exosomal secretion, at least in this specific context. Taking into account these findings, we next tested LUCAT1 and CASC9 serum and serum exosomes levels in 14 patients from our validation cohort, selected on the basis of serum exosomes availability, by using the same protocols applied to miRNAs isolation from whole serum and serum exosomes [17]. These analyses showed that the circulating amount of CASC9 and, at a lesser extent, LUCAT1 is relevant when compared to the matched HCC tissue (Figure 7B, 7C). The larger fraction of circulating LUCAT1 and CASC9 can be found in exosomes, further confirming the relevance lncRNAs as possible non-invasive biomarkers. Remarkably, serum CASC9 levels directly correlated with HCC nodule size (Pearson's correlation 0.54, p=0.04) indirectly supporting their secretion from tumor mass into the bloodstream. An inverse correlation was found between serum and tissue CASC9 expression (Pearson's correlation -0.531, p=0.05), while no correlation was found in the case of LUCAT1, which, in comparison with CASC9, displayed lower circulating and tissue levels. Since higher LUCAT1 and CASC9 tissue expression was associated with a lower recurrence rate, we further explored their circulating levels in patients with different recurrence rate, to assay their possible role as prognostic non-invasive biomarkers. Serum CASC9 and LUCAT1 were tested in 40 HCC patients at surgery. Circulating CASC9 levels turned out to be higher in patients with a disease recurrence within two years when compared to patients without recurrence at two years (Figure 7D). Conversely, we did not observe any difference in LUCAT1 circulating levels (Figure 7E) as well as in miR-181d and c circulating levels. These findings deserve further investigation on the possible role of CASC9 as a non-invasive prognostic biomarker, since our data suggest that HCCs with lower tissue expression and higher circulating levels of CASC9 more frequently develop metastasis after surgery. Finally, CASC9 and LUCAT1 serum levels were assayed in cirrhotics with (N=28) and without HCC (N=14) and no significant difference was found, ruling out their possible role as diagnostic non-invasive biomarkers (Figure 7F, 7G).

## DISCUSSION

Over 68% of genes in the human transcriptome were classified as lncRNAs, which are non-coding transcripts longer than 200 nucleotides [18]. Gene expression profile-based prognostic and therapeutic classifications have been proposed to characterize different subgroups of HCC, however, accumulating evidences on the roles of non-coding RNAs, make it mandatory to consider this class of RNAs also, to better classify tumors. Several functions have been identified for lncRNAs including the recruitment of chromatin-remodeling complexes to specific genomic loci that modify histone and RNA polymerase accessibility, miRNAs-sponge binding, and multiple epigenetic changes. As an example, in HCC, the lncRNA TUG1 was shown to regulate the Hedgehog pathway by targeting miR-132 [19]. The LncRNA 657 was demonstrated to suppress HCC cell growth by sponging miR-106a-5p [20] and MALAT1 promotes HCC progression by down-regulating miR-146b-5p through its sponge function, thereby promoting cancer growth and metastasis [21].

As relevant factors modulating tumor cell growth, apoptosis, invasion potential, lncRNAs have been proposed as diagnostic and prognostic biomarkers as well as putative therapeutic targets [22].

Here we profiled lncRNA expression in HCC and cirrhosis by using a RNA-sequencing approach. Data from the discovery set were validated in an independent series of patients and clinical-pathological characteristics were compared according to lncRNAs expression. Among deregulated lncRNAs, CASC9 and LUCAT1 were further validated due to the extent of their up-regulation in half of HCCs, while LINC01093 was chosen due to the extent of its down-regulation in HCC tissue. LUCAT1 (Lung Cancer Associated 1) is located at chromosome 5q14.3. It is transcribed in the opposite direction with respect to its closest protein coding genes, ADGRV1 (Adhesion G protein-coupled Receptor V1) encoding a member of the G-protein coupled receptor superfamily, and ARRDC3 (Arrestin Domain Containing 3), a member of the Arrestin family of proteins, which regulate G protein-mediated signaling.

CASC9 (Cancer Associated candidate 9) is located at chromosome 8q21.13 and it is transcribed in the opposite direction with respect to the closest protein coding genes HNFG4 (Hepatocyte nuclear factor 4 gamma) and CRISPLD1, whose functions are still poorly known. LINC01093 is a long intergenic non-protein coding RNA, located on chromosome 4q35.1 whose functions are still largely unknown. In our series of surgically resected HCCs, half of the cases displayed a concordant up-regulation of LUCAT1 and CASC9 which was associated with a lower recurrence rate after surgery. This appeared to be quite surprising since previous evidences reported LUCAT1 up-regulation to correlate with poor prognosis in NSCLC. Similarly, CASC9, which is up-regulated in esophageal cancer tissue, suppressed cell migration and invasion *in vitro* in knockdown experiments in that cancer type [23]. In line with these findings, Su et al. identified CASC9 increased expression as a factor facilitating cell growth and correlated with a poor prognosis of naso-pharyngeal cancer patients [24]. Recently, Klingenberg et al identified CASC9 among the lncRNAs up-regulated in HCC and affecting cell viability, proliferation and apoptosis [25]. Remarkably, in that study, CASC9 up-regulation was associated with a shorter OS in a subgroup of HCC patients of the TCGA cohort. In our series, which is smaller and not fully reliable for survival analysis due to heterogeneous treatments of post-surgical recurrence, we could not confirm data on OS. In addition, differently from Klingenberg et al, we could not observe any difference in the proliferation rate following CASC9 inhibition in HCC cells. Indeed, the discordant expression profiles of non-coding RNAs is a matter of fact in different tumor types and in different subgroups of specific cancer types, as widely demonstrated for microRNAs. Numerous microRNAs display a discordant deregulation pattern in different tumors, as well as an oncogenic or tumor-suppressive functions in different tumor types. Thus, the same situation might apply to lncRNAs with tissue- and cell-specific roles of these molecules, as previously reported by Tsoi and coworkers [26].

For these reasons we focused on CASC9 and LUCAT1 dependent molecular mechanisms contributing to HCC recurrence and functional experiments were performed in HCC-derived cell lines, confirming that the inhibition of both LUCAT1 and CASC9 increases migration capability and MMP9 expression. In line with these findings, an up-regulation of both LUCAT1 and CASC9 is associated with a lower recurrence rate and with a longer TTR in patients surgically resected for HCC. This observation was not restricted to HCC, but it was also confirmed in cirrhotic tissue of patients surgically resected for HCC. Remarkably, CASC9 and LUCAT1 up-regulation applies to 50% of HCCs only, thus this event cannot be considered a molecular feature of all HCCs. Instead, it might characterize a definite subgroup of tumors which, in our study, displays a lower aggressiveness. Understanding the specific mechanisms through which these LNCRNAs drive carcinogenesis still remains a challenge. Since the lncRNAs tested here are not conserved among the different species, we could not test an animal model of HCC, thus we limited the functional characterization of LUCAT1 and CASC9 *in vitro* and in primary tumors. A correlation between LUCAT1 and CASC9 deregulated expression and EMT-related biomarkers such as CDH1 and CDH2 also corroborated the findings that overexpression of these lncRNAs is associated with a lower migration potential as well as a “epithelial” phenotype in HCC. However, no correlation was found between CASC9 and LUCAT1 expression and other factors involved in EMT, such as TWIST, ZEB1, ZEB2, SNAIL1, SNAIL2, VIM. In addition, any interplay between CASC9 and LUCAT1 with the wnt-beta-catenin pathway was ruled out, by testing possible correlations with CTNNB1 expression in primary HCCs and in manipulated cell lines as well as with TCF7L2 in primary tumors. Thus, these data do not allow definitive correlations between LUCAT1 and CASC9 deregulated expression and modulation of specific EMT factors, with the exception of CDH1 and CDH2.

Since LNCRNAs mainly act as modulators of other classes of RNAs, we thus focused on their sponging functions. A possible mechanism explaining the association between higher LUCAT1 expression and lower HCC recurrence rate, was the sponging effect exerted by this LNCRNA on miR-181d-5p. This event was assessed by the luciferase reporter assay and its relevance was confirmed by LUCAT1 an miR-181d-5p inverse correlation in primary HCCs. Indeed, miR-181d-5p was previously demonstrated to maintain an undifferentiated state of hepatic progenitor cells contributing to the “stemness” phenotype and its overexpression is associated with a more aggressive subgroup of HCCs being up-regulated in hepatic stem cell populations and in HCC cells with progenitor features [27]. In addition, miR-181 family was recently shown as up-regulated in an aggressive subgroup of HCCs [15]. Thus, it is conceivable that the sponge function exerted by LUCAT1 on miR-181d-5p, might contribute to reduce miR-181d-5p levels in HCCs overexpressing LUCAT1 thus lowering the invasion potential of this subgroup of tumors (Supplementary Figure 4). Indeed, we cannot rule out the possibility that the lower recurrence rate ascribed to LUCAT1 and CASC9 up-regulation is instead due to miR-181 down-regulation. Long non-coding RNAs participate to carcinogenesis due to their function of regulatory elements. Beside LUCAT1-miR-181 interaction, several other predicted interactions with other miRNAs can be identified by bioinformatics algorhytms for both LUCAT1 and CASC9. The validation of the most relevant ones will allow the discover of driving mechanisms in each specific context.

High circulating levels of CASC9 were found in patients with increased recurrence rate. These data let us to hypothesize a role of extracellular secretion of these lncRNAs as previously demonstrated for miR-221 in Sorafenib treated HCCs [28]. We thus tested whether CASC9 and LUCAT1 were secreted in exosomes confirming their consistent exosomal secretion in HCC-derived cell lines and in a preliminary series of patients. Indeed, lncRNAs protection by exosomes extracted from serum of patients with non-small cell lung cancer was previously reported [16], and confirmed in our HCC patients suggesting the relevance of lncRNAs as putative circulating biomarkers. The lncRNAs population isolated from serum does not necessarily derive from tumor cells. Indeed, other cell and tissue types can contribute to serum lncRNAs, including immune system derived cells, inflammatory and stromal tissues. However, irrespective from the causative mechanisms and cells of origin, the increased serum CASC9 levels in patients with larger HCCs and higher recurrence rate, suggest its further investigation as a putative prognostic biomarker, as previously reported for MALAT1 [11]. Thus even though our data did not indicate serum CASC9 as a promising diagnostic biomarker, it appears to be a good candidate prognostic biomarker to be further exploited in prospective studies in patient with HCC. The small cohort and its retrospective nature, far from optimal for clinical and prognostic correlates such as survival predictions, the high molecular heterogeneity, the still poorly known functions of lncRNAs, represent relevant limitations of this study. Understanding the roles and phenotypic effects of lncRNAs disruption in specific diseases gains prominence, and even though very preliminary, our study underlies the aberrant expression of a panel of lncRNAs in HCC, outlining their possible exploitation as non-invasive biomarkers of recurrence.

## MATERIALS AND METHODS

### Patients

Matched HCC and cirrhotic tissues were obtained from 60 patients (46 males and 14 females) undergoing liver resection for HCC. In detail, two cohorts were independently examined in a discovery and validation setting by using different analytical approaches: lncRNAs were profiled by RNA-sequencing in a discovery set of 28 matched HCC and cirrhotic tissues. Data were confirmed by Real Time RT-PCR in the same series. An independent series of 32 matched HCC and cirrhotic tissues (validation set) was analyzed by Real-Time RT-PCR to verify the deregulated expression of the most significant lncRNAs (LUCAT1, CASC9, LINC01093) emerged from the discovery phase. Both series were selected to include two different groups in terms of outcome: the first group included patients with recurrence within 24 months after surgery while the second group included patients without recurrence at two years after surgery. Patients characteristics are reported in Supplementary Table 1. Tissues were collected at surgery and stored as previously described [29]. Histopathological grading was scored according to Edmondson and Steiner criteria [30]. No patient received anticancer treatment prior to surgery. Antiviral treatment was administered to HBV-infected patients only. No HCV-infected patient received antiviral treatment except for interferon-based regimens performed at least three years before surgery. In 14 of 32 cases of the validation series, serum samples were also collected and examined for circulating lncRNAs expression. These patients were tested for lncRNAs serum/tissue correlations and circulating levels of lncRNAs in these 14 patients with cirrhosis complicated by HCC were compared with an independent series of 14 cirrhotic patients without HCC. An additional cohort of 11 patients undergoing orthotopic liver transplantation for end-stage liver disease without HCC and 10 healthy liver tissues undergoing surgery for haemangiomas or traumatic liver lesions were assayed by Real-Time RT-PCR for LUCAT1, CASC9 and LINC01093 expression.

### RNA sequencing and bioinformatics analysis

Total RNA was extracted from tissue samples of HCC and adjacent cirrhosis, from 60 patients undergoing surgery, from11 patients undergoing OLT for cirrhosis without HCC and from 10 healthy liver using TRIzol (Invitrogen, Life Technologies, Carlsbad, CA) according to the manufacturer's instructions. The concentration and purity of the RNA samples were determined using Nanodrop spectrophotometer (Thermo-fisher Scientific, Rodano, Italy). Twenty-eight cases were studied by RNA-sequencing. The libraries for whole transcriptome sequencing were prepared using 1 ug of total RNA by using TruSeq RNA Sample Prep KIT v2 (Illumina Inc. San Diego, USA) according to the manufacturer's instructions. Libraries concentration was assessed with Picogreen (Invitrogen), while quality control and average size of prepared libraries were performed using the Agilent Bioanalyzer (Agilent, Santa Clara, CA). Whole transcriptome sequencing was performed on Illumina HiScanSQ platform adopting the paired-end strategy (2×80 bases). All 28 HCC/cirrhosis samples were sequenced together for a total of 400 GB (Giga bases) and 95×10^-6^ average reads per sample and an average coverage of 61X. The FASTQ raw sequencing data were generated with the bcltofastq function (provided by Illumina) and AdapterRemoval (https://github.com/MikkelSchubert/adapterremoval) was adopted to trim stretches of low quality bases (< Q10) and to remove Truseq adapters. The paired-end reads were mapped with TopHat/BowTie pipeline (http://ccb.jhu.edu/software/tophat) using the human genome HG19 as reference (http://genome.ucsc.edu), PCR and optical duplicates were removed with Samtools (http://samtools.sourceforge.net). Transcripts abundance (Ensembl release 72 annotation features) was calculated adopting the function htseq-count (Python package Htseq – https://htseq.readthedocs.io/en/release_0.10.0/), the R-Bioconductor package edgeR (https://bioconductor.org/) was applied to normalize the expression data. Data from RNAseq experiment were analyzed with GeneSpring GX v.14 (Agilent Technologies). Specifically, counts were imported in GeneSpring software as a custom expression technology. HCC and LC data were log2 transformed. Differentially expressed non-coding genes were identified using paired t-test with a fold change > 2.0 and an adjusted p<0.05, for HCC vs LC comparison, Benjamini and Hochberg correction was applied to adjust p-values for multiple testing. Supervised hierarchical clustering for long non-coding RNAs was performed using differentially expressed genes and Manhattan correlation as a distance measure.

### Real-time PCR and reverse transcription (RT)-PCR

For the validation purposes, both the discovery and the validation series were examined for LUCAT1, CASC9 and LINC01093 expression by Real-Time RT-PCR, as previously described [31, 32]. The same protocol was used for the serum expression analysis of lncRNAs. PCR primers and annealing temperature are listed in Supplementary Table 2. Gene expression of specific factors involved in epidermal to mesenchymal transition was quantified by a Custom RT^2^ Syber green semi-quantitative PCR. All reagents were purchased from Qiagen (Hilden, Germany). All Real-Time RT-PCR experiments were run in triplicate and GADPH was used as housekeeping gene for tissue assays, while beta-actin was used for serum and serum exosomes assays. For circulating/tissue lncRNAs correlations, beta actin was used as housekeeping for normalization of both serum and tissue expression. 2^-ΔΔCt^ method was used to quantify lncRNAs in tissues, serum and serum exosomes. Conventional RT-PCR was used to verify gene silencing and for the detection of LUCAT1 and CASC9 expression in exosomes isolated from culture media of SNU449 and PLC/PRF5 cells. The expression of mature miRNAs in HCC tissues was assayed using the Taqman MicroRNA Assays (Thermofisher) specific for miR-181c-5p (code 000482), miR-181d-5p (code 001099) and miR-145-5p (code 002278) on 60 matched HCCs and cirrhosis as previously described [29]. 2^-ΔΔCt^ method was used to quantify miRNA, lncRNA and mRNA expression. RNU6B (code 001973) and beta-actin were used as reference genes respectively for miRNAs and LNCRNAs assays. Each sample was analyzed in triplicate.

### Cell culture and treatments

HepG2, Hep3B, SNU398, SNU449, SNU182, SNU475 and PLC/PRF5 cell lines were obtained from American Type Culture Collection (ATCC, Rockville, MD, USA) and maintained according to ATCC instructions. Huh-7 cells are from Prof. Alberti's laboratory, University of Padua, Italy and cultured with RPMI-1640 (Life Technologies). Media were supplemented with 10% FBS, 2 mm L-Glutamine and 1X Penicillin/Streptomycin (Life Technologies). All experiments were performed in triplicate.

### Cell transfection

HCC cells were seeded into 6 well plates and transfected with 20 nM of CASC9, LUCAT1 (IDT, Thief River Falls, MN, USA), siRNAs or scrambled siRNA (scRNA) using Lipofectamine2000 (Invitrogen) according to manufacturer's instructions. Transfection efficiencies were greater than 90% as determined by co-transfection with a fluorescein-labelled siRNA (Invitrogen). Analyses were performed 24h and 48h post-transfection.

### Dual-luciferase reporter assay

A portion of LUCAT1 lncRNA, containing miR-181d hypothetical binding site, was amplified by using the following primers: Fw 5’-TGTGCTCTAGATGCTGTTGATG-3’, Rv 5’CAACTCTAGAGGCACGCTAAG-3’and cloned into pGL3-control vector at the XbaI site. Dual-luciferase reporter assay (Promega) was performed in HepG2 cells as previously described [33]. MiR-181d-5p was chosen among miR-181 family members due to the inverse correlation between miR-181d-5p and LUCAT1 expression in primary HCCs.

### FACS analyses

Cell cycle analysis in HCC cells silenced for LUCAT1 and CASC9 was performed in triplicate by flow cytometry (FACSaria cell sorter, BD Biosciences) as previously described [34].

### Cell viability assay

The CellTiter luminescent viability assay was used to determine the number of viable cells in culture based on quantitation of ATP, which signs the presence of metabolically active cells. CellTiter assay was performed according to Promega instructions in control and transfected cells (Promega, Madison, WI, USA).

### Invasion assay

Invasion assay in HCC-derived cell lines manipulated for LUCAT1 and CASC9 expression was performed as previously described by Boyden blind-well chambers containing poly-vinyl-pyrrolidone–free polycarbonate filters, 8-μm pore size coated with Matrigel (Sigma, Saint Louis, MO, USA)[35]. Invasive cells were fixed with 4% paraformaldehyde, stained with Giemsa (Sigma), and counted under a microscope.

### Immunocytochemistry (ICC)

Cells were seeded on sterilized coverslips and fixed in cold methanol. Cells were then incubated with normal goat serum at RT for 30 min. Beta-Catenin protein localization was assessed by using a BD monoclonal antibody diluted 1:400 followed by a HRP-rabbit EnVision system with diaminobenzidine (DAB) (Sigma) as chromogen. Cells were then counterstained with Mayer's hematoxylin and mounted with DPX (BDH Chemical, Poole, UK). Negative controls were obtained by omitting the primary antibody.

### Serum and exosomes lncRNAs determinations

Isolation of lncRNAs from whole serum, serum exosomes and cell culture supernatant including the exosomal fraction of control and manipulated cells was performed by an ultracentrifugation-based protocol as previously reported for circulating miRNAs [17]. Briefly, exosomes isolation from 400 μl of serum was executed by ultracentrifugation at 100.000 × g for 70 min at 4°C. Exosomes pellet obtained by ultracentrifugation was washed in PBS and collected by ultracentrifugation as above. Final pellets containing exosome vesicles were suspended in 400 μl of PBS. The same procedure was applied to exosomes isolation from cell culture supernatants. Cells were collected from the same flasks used for the detection of extracellular exosomes. Trizol Reagent (Life Technologies) was used to isolate mRNAs (namely beta-actin used as an housekeeping for lncRNAs) and lncRNAs (CASC9 and LUCAT1). MiRNeasy kit (miRNeasy Serum/Plasma Kit, Qiagen) was used for microRNAs isolation. For serum miRNAs assays, Cel-miR-39 (Ambion), a synthetic 21-mer RNA (12.5 fmol) which sequence does not match any known human small RNA sequence, was added to serum samples and was used for normalization of qPCR data (Taqman microRNA assays, Life Technologies specific for each miRNA). Each qPCR reaction was run in triplicate and the average expression levels were calculated as previously described [17] by using the 2(-ΔΔCt) method.

### Statistical analysis

Data elaborations for the RNA-sequencing study were detailed above. Concerning Real Time RT-PCR data and data from *in vitro* experiments, results were expressed as mean ± SD. Normally distributed data were compared by two-tailed Student's t-test (for comparison of two groups) and one-way ANOVA (for comparison of multiple groups). Non-normally distributed data were analyzed by Mann-Whitney U test (for comparison of two groups). Pearson's correlation coefficient was used to explore relationships between variables. Associations between lncRNAs deregulated expression and clinical-pathological features of HCCs including age, gender, etiology, serum AFP, liver function, grading and histopathological characteristics of HCC, micro and macrovascular invasion, staging and nodularity, recurrence after surgery, were tested by Student's t-test and Pearson's correlation. For survival variables such as time to recurrence and OS, Kaplan-Meier curves and parameters were derived. Time to recurrence (TTR) was evaluated starting from the time of surgery, based on mRECIST tumor assessment. Patients were categorized on the basis of lncRNAs median expression values in HCC and cirrhotic tissues. The stepwise backward selection model was performed to assay variables independently associated with HCC recurrence, including CASC9, LUCAT1 and LINC01093 levels. *In vitro* experiments were all performed in triplicate and the mean values were used for the statistical analysis. Reported p-values were two-sided and considered significant when lower than 0.05. Statistical calculations were executed using SPSS version 19.0 (SPSS inc). ^*^p<0.05, ^**^p<0.01, ^***^p<0.001.

## SUPPLEMENTARY MATERIALS FIGURES AND TABLES



## Abbreviations

HCC: hepatocellular carcinoma
LC: liver cirrhosis
ncRNA: non-coding RNA
lncRNA: long non-coding RNA
mRNA: messenger RNA
LUCAT1: Lung Cancer Associated 1
CASC9: Cancer Associated Candidate 9
OLT: orthotopic liver transplantation
