# Supplementary material for: LncRNAs as novel players in hepatocellular carcinoma recurrence
Source: Oncotarget. 2018 Oct 12;9(80):35085–99. doi: 10.18632/oncotarget.26202 (PMC6205555; doi:10.18632/oncotarget.26202)
Supplement: Supplementary file 1 [file oncotarget-09-35085-s001.pdf]

# LncRNAs as novel players in hepatocellular carcinoma recurrence

## SUPPLEMENTARY MATERIALS

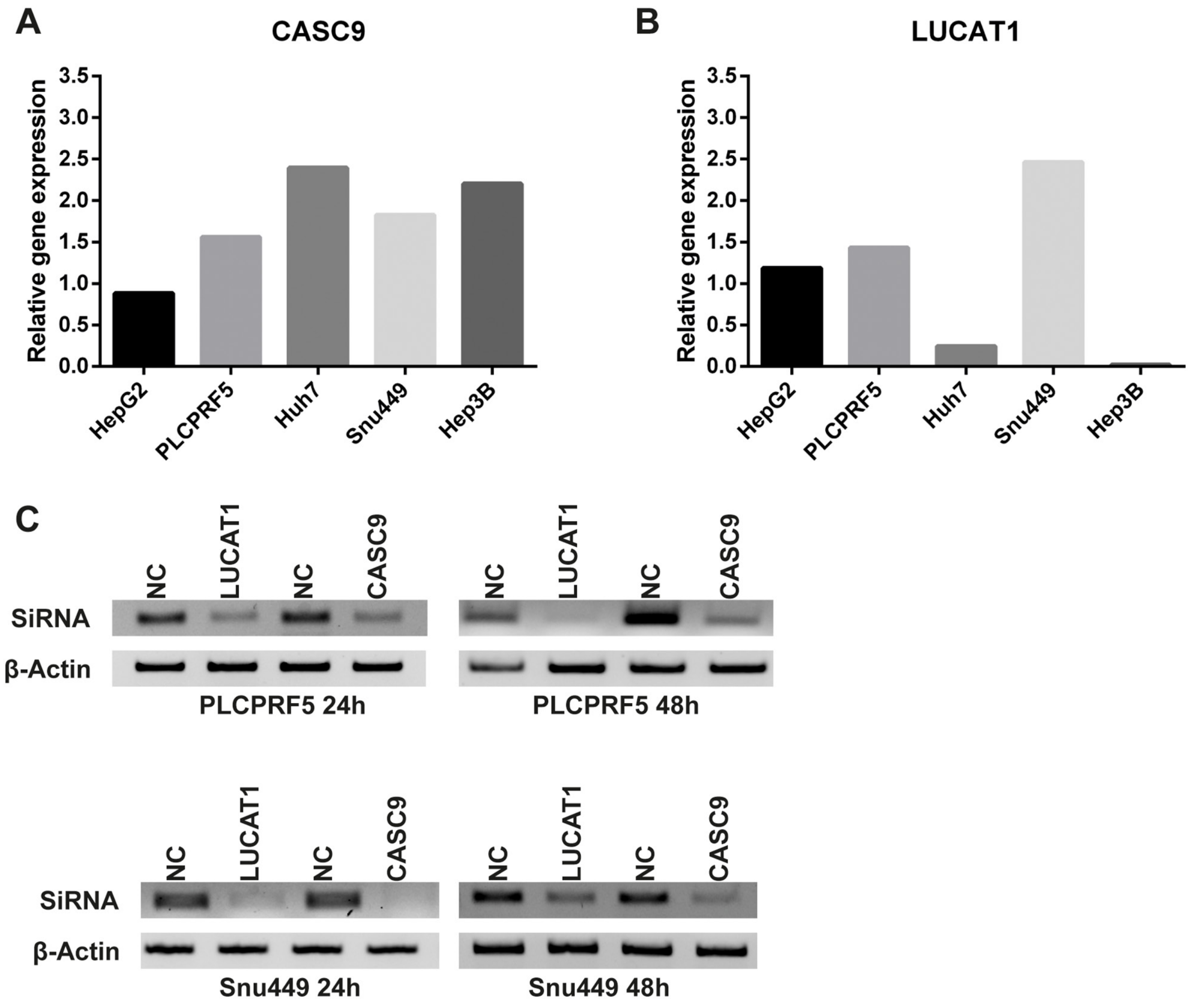


**Supplementary Figure 1: Long non-coding RNAs expression and silencing in HCC cell lines.** **(A-B)** Real-Time PCR analysis of CASC9 and LUCAT1 in different HCC cell lines. Y-axes reported $2^{-DDCt}$ values corresponding to CASC9 and LUCAT1 levels. **(C)** PLCPRF5 and SNU449 were transiently transfected with siRNAs directed against CASC9 and LUCAT1 or negative control (NC) for 24h and 48h. CASC9 and LUCAT1 silencing was evaluated by conventional PCR. β-Actin was used as housekeeping gene.

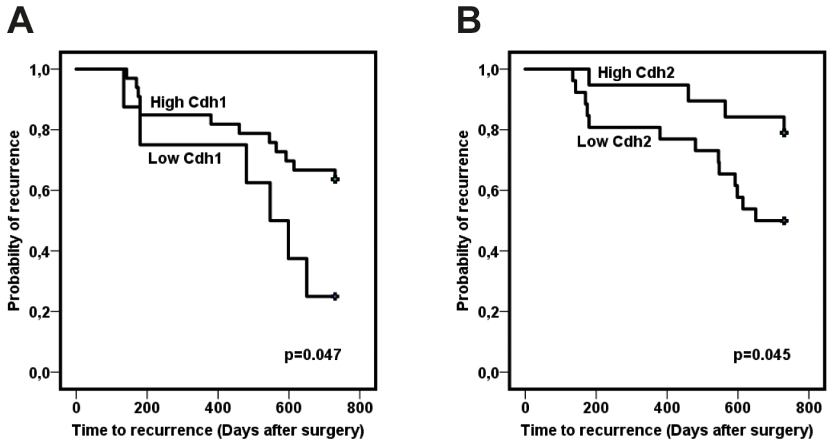


**Supplementary Figure 2: Role of Cdh1 and Cdh2 in HCC recurrence after surgery. (A-B)** Association between Cdh1 and Cdh2 levels and TTR of surgically resected HCC patients. High and low, cdh1 and Cdh2 expression was categorized according to the mean value. Log-rank P values are from Kaplan-Meier analysis.

A

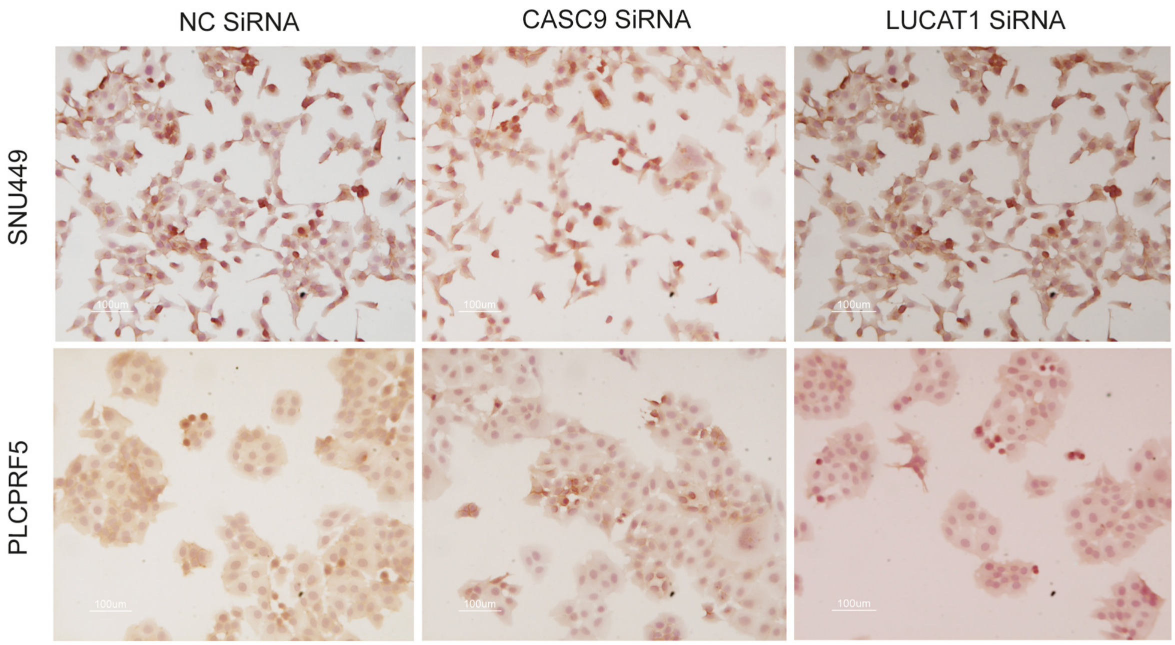


**Supplementary Figure 3: Beta-Catenin detection by immunocytochemistry.** Expression and subcellular localization of Beta-Catenin, as detected by the immunoperoxidase method, of the SNU449 and PLCPRF5 cell lines. Positive staining was observed in the nucleus and at membrane level. Nuclei were counterstained with hematoxylin. Original magnification 20X.

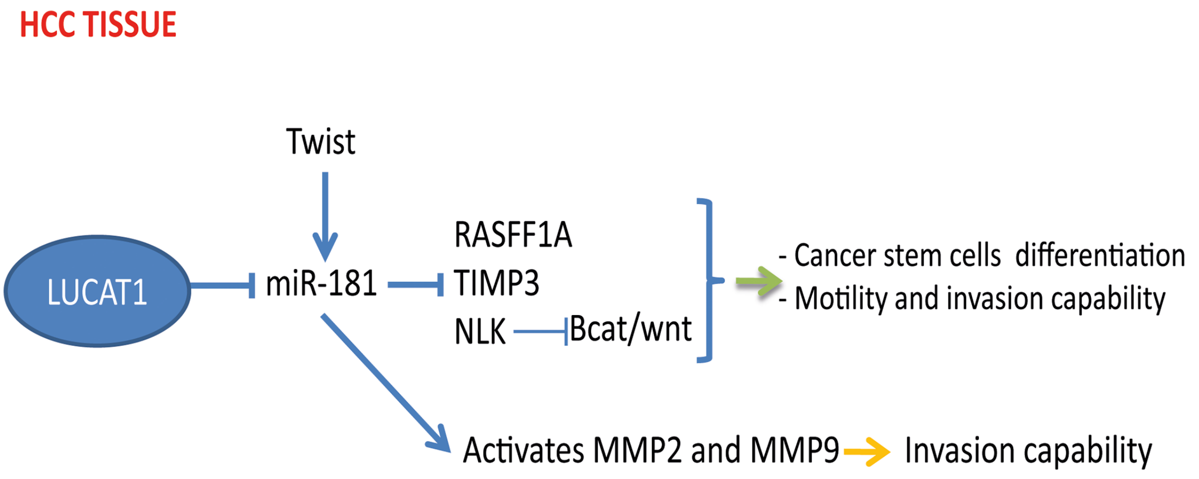


**Supplementary Figure 4: Possible mechanisms downstream LUCAT1-miR-181 interaction.** Possible mechanisms downstream miR-181d sponging by LUCAT1 in EMT and invasion modulation.

**Supplementary Table 1: Patient characteristics - discovery (N=28) and validation (N=32) cohorts**

| Factors | | Frequency(%) Discovery - 28 pts | Frequency(%) Validation - 32 pts |
|---|---|---|---|
| Etiology | HBV | 4/28 (14.3%) | 8/32 (32%) |
| | HCV | 18/28 (64.3%) | 15/32 (46.9%) |
| | Alcohol abuse | 2/28 (7.1%) | 3/32 (9.4%) |
| | None | 4/28 (14.3%) | 6/32 (18.7%) |
| Cirrhosis | | 24/28 (85.7%) | 27/32 (84.4%) |
| Serum AFP | > 20 ng/ml | 11/28 (39.3%) | 18/32 (56.2%) |
| Edmondson grade | I | 0/28 (0%) | 1/32 (3.1%) |
| | II | 12/28 (42.8%) | 11/32 (34.4%) |
| | III | 14/28 (50%) | 16/32 (50%) |
| | IV | 2/28 (7.2%) | 4/32 (12.5%) |
| Barcelona Clinic Liver Cancer stage | O | 1/28 (3.6%) | 2/32 (6.3%) |
| | A | 25/28 (89.3%) | 26/32 (81.2%) |
| | B | 2/28 (7.1%) | 4/32 (12.5%) |
| | C | 0% | 0% |
| | D | 0% | 0% |

**Supplementary Table 2: Primer sequences for RT-PCR**

| Gene | Primers sequence (5'-3') | Annealing T (°C) | Product size (bp)* |
|---|---|---|---|
| CASC9 F†<br>CASC9 R ‡ | GCCACATTCATGGTGTTGAG<br>TCTTGCCAGGTGTTGTTCTG | 60 | 77 |
| LUCAT1 F<br>LUCAT1 R | ACCCAGGAATCCAACTTGC<br>TCACATTCAGCCCCTTTAGC | 60 | 80 |
| LNC01093 F<br>LNC01093 R | AAGAATGGAAGCCCTGGAAG<br>ACAGCAGTGGGGAATCTTTG | 60 | 164 |
| GADPH F<br>GADPH R | GAGTCAACGGATTTGGTCGT<br>GACAAGCTTCCCGTTCTCAG | 60 | 185 |
| β-ACTIN F<br>β-ACTIN R | GAGGCACTCTTCCAGCCTTC<br>GGATGTCCACGTCACACTTC | 60 | 189 |

* bp, base pairs; † F, forward; ‡ R, reverse
